# Central Composite Design Implemented Azilsartan Medoxomil Loaded Nanoemulsion to Improve Its Aqueous Solubility and Intestinal Permeability: In Vitro and Ex Vivo Evaluation

**DOI:** 10.3390/ph15111343

**Published:** 2022-10-29

**Authors:** Girish Kumar, Tarun Virmani, Kamla Pathak, Omkulthom Al Kamaly, Asmaa Saleh

**Affiliations:** 1 School of Pharmaceutical Sciences, MVN University, Haryana 121105, India; 2 Faculty of Pharmacy, Uttar Pradesh University of Medical Sciences, Etawah 206001, India; 3 Department of Pharmaceutical Sciences, College of Pharmacy, Princess Nourah bint Abdulrahman University, P.O. Box 84428, Riyadh 11671, Saudi Arabia

**Keywords:** azilsartan medoxomil, nanoemulsion, aqueous solubility, bioavailability, central composite design, optimization

## Abstract

The present research attempted to design and develop a nanoemulsion formulation of azilsartan medoxomil to improve its aqueous solubility and intestinal permeability. Based on the solubility profile, ethyl oleate, tween 80, and Transcutol P were selected as the oil phase, surfactant, and co-surfactant, respectively. Central composite design (CCD) suggested an optimized azilsartan medoxomil- nanoemulsion formulation (optimized AZL-NE formulation) with 1.25% oil, 15.73% Smix, and 90 s ultrasonication time; it was found to have the droplet size, percentage transmittance, and % cumulative drug release (%CDR) of 71.5 nm, 93.46 ± 1.13%, and 90.14 ± 0.94%, respectively. Furthermore, it exhibited a 0.141 polydispersity index, 34.05 mV zeta potential, a 1.413 ± 0.03 refractive index, 6.68 ± 0.22 pH, 28.17 ± 0.52 cps viscosity, and a 96.98 ± 0.94% percentage drug content. Transmission electron microscopy (TEM) assessed the nano-sized spherical shape, and a differential scanning calorimeter (DSC) assessed the solubilization of the drug in the optimized formulation. The %CDR was 1.71 times higher and the % cumulative drug permeation was 2.1 times higher for the optimized AZL-NE formulation than for the drug suspension through an intestinal segment of a rat, which was also supported by confocal laser scanning microscopy (CLSM) studies. Thus, the nanoemulsion formulation of azilsartan medoxomil ensured the enhancement of the drug availability in the body.

## 1. Introduction

Azilsartan medoxomil (AZL) is one of the highly selective angiotensin II AT_1_ receptor antagonists; it exhibits antihypertensive activity due to the blocking of the direct vasoconstriction caused by the angiotensin II enzyme [1]. It has a molecular weight of 568.54 g/mol along with the ionization constant (pKa) and partition coefficient (log P) values of 6.1 and 4.9, respectively [2]. It falls under the BCS class II category as it has the aqueous solubility of 0.00978 mg/mL at 37 °C, which also leads to its poor dissolution properties and low bioavailability of 60% [3]. Poor aqueous solubility and low bioavailability present the main hindrances to the therapeutic efficiency of azilsartan medoxomil in the treatment of hypertension. It is a prodrug that is hydrolyzed to the azilsartan in the gastrointestinal tract during the absorption phase, and its absorption is not affected by the presence of food. The maximum drug plasma concentration was achieved within 1.5 to 3 h. It is 99% protein-bound and has a half-life of 11 h [4]. Despite poor aqueous solubility and low bioavailability, limited drug delivery systems have been prepared to improve the aqueous solubility and bioavailability of azilsartan medoxomil, including solid dispersions [5], nanocrystals [3], hydrotrophy, and nanosuspension [2]. It shows that various novel drug delivery systems, such as nanoemulsion, solid lipid nanocarriers, nanostructured lipid carriers, dendrimers, and polymeric micelles, are unexplored for their potential to improve the aqueous solubility and bioavailability of azilsartan medoxomil.

Nanoemulsion was selected as a drug delivery system due to its possession of various advantages, such as improved aqueous solubility, enhanced permeability, improved stability, reduced adverse effects leading to higher efficacy, encapsulation of both hydrophilic and hydrophobic drugs, ease of manufacturing, and relatively economical and improved bioavailability [6]. The methods used to prepare the nanoemulsion have been categorized into two categories, namely high-energy methods and low-energy methods. The high-energy methods include microfluidization, ultrasonication, and homogenization, whilst the low-energy methods include spontaneous emulsification, phase inversion temperature, and phase inversion composition [7]. Various factors, including oil concentration, S_mix_, and timing of the process, are crucial parameters in the preparation of nanoemulsion, which must be optimized to provide the formulation with better results [8]. The optimization of these parameters can be obtained using various optimization techniques, such as the central composite design (CCD), the Box–Behnken design (BBD), the full-factorial design, and the Doehlert matrix (DM), but in pharmaceutical research, CCD and BBD are mainly employed to optimize various independent variables. Moreover, CCD has an advantage over BBD due to the possession of an extra edge, which provides much better predictions. BBD suggests that the formulations have a low, medium, and high value of variables, whilst CCD provides two more extreme values, namely +α and −α which give a rotability to the design [9].

## 2. Results

### 2.1. Selection of Formulation Components Using Solubility Determination

**Figure 1:**
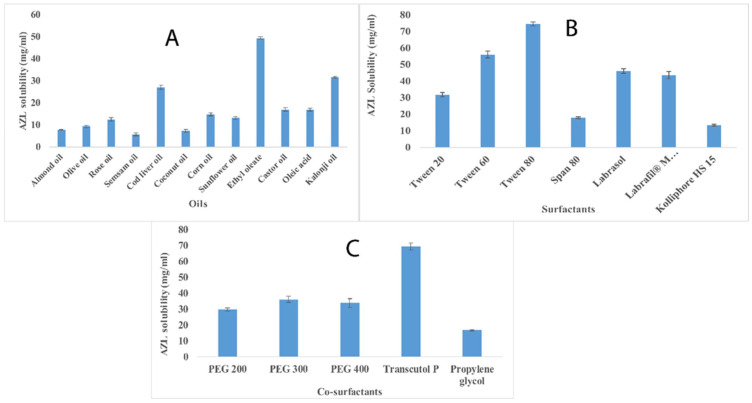
(**A**–**C**) depict the solubility of azilsartan medoxomil in various oils, surfactants, and co-surfactants.

The solubility profile of AZL in various oils, surfactants, and co-surfactants is given in Figure 1. The solubility of AZL in various oils was in the order of ethyl oleate (49.32 mg/mL), Nigella sativa oil (32.33 mg/mL), cod liver oil (28.81 mg/mL), castor oil (16.55 mg/mL), oleic acid (16.45 mg/mL), corn oil (15.18 mg/mL), sunflower oil (12.55 mg/mL), rose oil (9.85 mg/mL), olive oil (9.41 mg/mL), coconut oil (6.66 mg/mL), and semsam oil (4.37 mg/mL) (Figure 1A). The oil is a requisite component in the formulation of nanoemulsion to dissolve the hydrophobic drug. However, the concentration of the oil must be as low as possible in the nanoemulsion formulation because the increase in the concentration of oil leads to an increase in the droplet size, which causes hindrances in the nanoemulsion formulation and a reduction in the physical stability of the prepared nanoemulsion [10]. Hence, the oil with the maximum solubility for a drug must be selected. The highest solubility of azilsartan medoxomil in ethyl oleate might be due to the presence of long-chain fatty acids in the structure of ethyl oleate. Moreover, ethyl oleate possesses improved solubilization, a higher nanoemulsion area, and efficacious lymphatic absorption [11]. Depending upon the solubility profile, ethyl oleate, Nigella sativa oil, and cod liver oil were selected as the oil phases for the miscibility studies.

A second important component in the formulation of nanoemulsion is a surfactant that improves the solubility of a drug by minimizing the interfacial tension between the oil phase and the water phase [12]. The surfactant was selected based on the solubility and miscibility studies. The solubility of azilsartan medoxomil in various non-ionic surfactants is depicted in Figure 1B. The order of solubility of AZL in various surfactants was tween 80 (72.27 mg/mL), tween 60 (52.5 mg/mL), labrasol (45.42 mg/mL), Labrafil^®^ M 1944 CS (43.67 mg/mL), and tween 20 (32.76 mg/mL) (Figure 1B). The highest solubility of AZL in tween 80 might be due to its greater capability to reduce the droplet size than the other surfactants owing to its low molecular weight [13]. Depending upon the solubility studies, tween 80, tween 60, and labrasol were selected as the surfactants for the miscibility determination.

Another important component in the formulation of the nanoemulsion is the co-surfactant, which is mainly added to improve the emulsification property provided by the surfactant or to reduce the concentration of the surfactant in the formulation [14]. The order of solubility of AZL in the various co-surfactants was Transcutol P (65.71 mg/mL), PEG 300 (32.28 mg/mL), PEG 400 (28.76 mg/mL), PEG 200 (28.23 mg/mL), and propylene glycol (17.55 mg/mL) (Figure 1C). Apart from the highest solubility of AZL in Transcutol P, it is also a good penetration enhancer [15]. Transcutol P, PEG 300, and PEG 400 were selected as co-surfactants for the miscibility determination based on the solubility profile.

### 2.2. Miscibility Determination of Oil, Surfactant and Co-Surfactant

**Figure 2:**
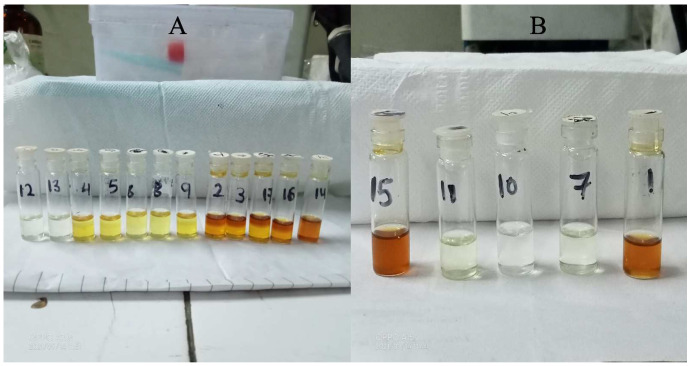
(**A**,**B**) depict the mixtures with phase separation and the clear mixtures, respectively.

The results of the miscibility determination are summarized in Table 1, which depicts that the mixtures having tween 80 as a surfactant and Transcutol P as a co-surfactant were miscible with all the selected oils except cod liver oil (Figure 2). Ethyl oleate, tween 80, and Transcutol P were selected as the oil phase, surfactant, and co-surfactant, respectively, to formulate the nanoemulsion because all three of these showed greater drug solubility while being miscible.

### 2.3. Pseudo-Ternary Phase Diagram Studies

**Figure 3:**
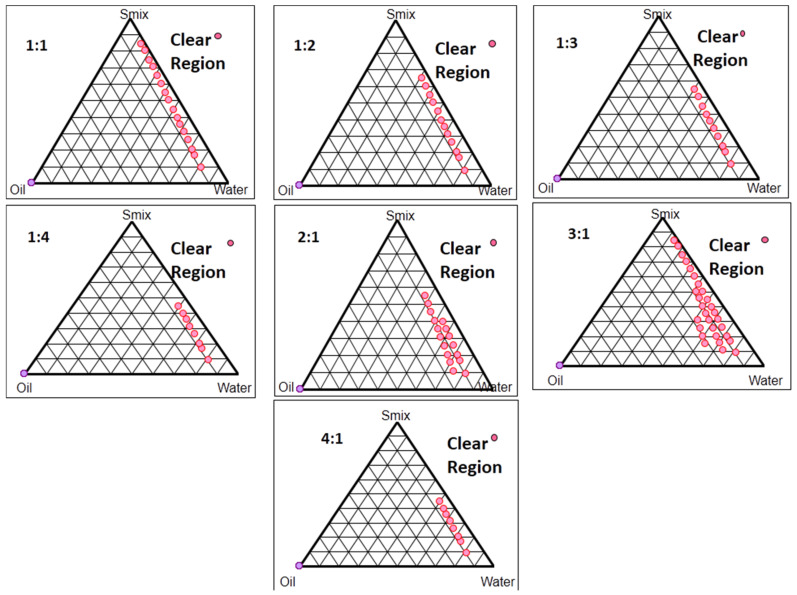
Pseudo-ternary phase diagrams depicting the existence of o/w nanoemulsion region (clear region) for different surfactants: co-surfactant ratios (or S_mix_).

The pseudo-ternary diagrams for every S_mix_ ratio are illustrated in Figure 3, which depicts the increase in the concentration of the co-surfactant in relation to the surfactant (1:1, 1:2, 1:3, 1:4); the region of the nanoemulsion formulation (clear region) was reduced, whilst there was an increase in the concentration of the surfactant in relation to the co-surfactant (2:1, 3:1, 4:1); the clear region was increased up to the S_mix_ ratio of 3:1, but after this, the increase in the concentration of the surfactant caused a reduction in the clear region (4:1). The enhancement of the clear region with the increase in the concentration of the surfactant might be due to the improved emulsification capacity with the increase in the concentration of surfactant (Tween 80) [16]. Moreover, the viscosity of the formulation also increased with an increase in the amount of tween 80, which could be the reason for the reduction in the region of nanoemulsion with the further increase in the concentration of tween 80, as shown by the diagram of S_mix_ 4:1, because the enhanced viscosity of the system harms the droplet distraction and break-up [13]. Based on these studies, the S_mix_ in the ratio of 3:1 and the oil: S_mix_ in the ratio of 1:7 showed the maximum region for the nanoemulsion formulation; hence, they were selected for further studies.

### 2.4. Optimization of Formulation Components for Nanoemulsion

A total of 17 different formulations suggested by CCD were prepared using the ultrasonication method, and the values for the droplet size (Y1), %transmittance (Y2), and %CDR (Y3) were in the range of 80.69–311.9 nm, 72.88–93.89%, and 22.06–94.92%, respectively. The experimental values and the predicted values for the dependent variables are summarized in Table 2. The optimization software proposed a polynomial quadratic (*p* = 0.0004 for Y1 and *p* < 0.0001 for Y2 and Y3) for all three of the dependent variables when the observed data for the dependent variables were submitted to it. The predicted R^2^ values for all three dependent variables were in agreement with the adjusted R^2^ values due to a difference of less than 0.2; these are summarized in Table 3 along with the mean, standard deviation, and coefficient of variation (%) for all the dependent variables. To predict the quantitative effects of all the independent variables with varying levels on the dependent variables, the software generated the polynomial equations for each response, which are the following:

Y1 (droplet size) = +96.63 + 42.31 × A − 44.24 × B − 22.97 × C − 11.14 × AB − 6.51 × AC + 21.05 × BC + 21.03 × A^2^ + 30.72 × B^2^ + 7.35 × C^2^ (1)

Y2 (%transmittance) = +91.88 − 3.58 × A + 5.08 × B + 1.11 × C + 1.81 × AB + 0.3000 × AC − 0.4350 × BC − 0.8776 × A^2^ – 3.36 × B^2^ − 0.7167 × C^2^ (2)

Y3 (% CDR) = +58.48 − 21.16 × A + 9.69 × B + 3.88 × C − 3.73 × AB − 4.63 × AC − 1.39 × BC − 0.09 × A^2^ − 7.03 × B^2^ − 0.7288 × C^2^ (3)

**Figure 4:**
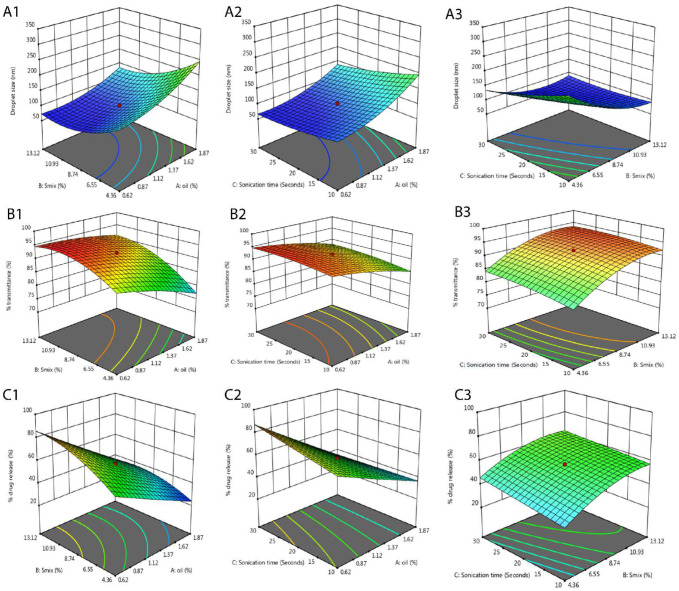
Three-dimensional surface plots depicting the interaction effects of independent variables on droplet size (**A1**–**A3**), % transmittance (**B1**–**B3**), and % CDR (**C1**–**C3**).

Equation (1) showed the impact of the oil concentration (A), S_mix_ concentration (B), and sonication time (C) on the droplet size of the formulation. The oil possessed a positive impact whilst the S_mix_ and sonication time possessed a negative impact on the droplet size, which means that with an increase in the oil concentration in the formulation the droplet size will increase, whilst it will decrease with an increase in the S_mix_ concentration and sonication time. The combination of oil*S_mix_ (AB) and oil*sonication time (AC) possessed a negative effect whilst S_mix_*sonication time (BC) possessed a positive effect on the droplet size, which was also supported by the 3D surface plots (Figure 4(A1–A3)) depicting that the increase in the concentration of the S_mix_ and the sonication time causes a decrease in droplet size whilst the increase in the concentration of oil causes an increase in the droplet size. The ANOVA analysis and the model summary statistics for the experimental results provided a greater R^2^ of 0.9853 for quadratic Equation (1).

By Equation (2) and Figure 4B, the % transmittance of the formulation was reduced with an increase in the concentration of oil, but it increased with an increase in the concentration of the S_mix_ and sonication time (Figure 4(B1–B3)). The combination of oil*S_mix_ and oil*sonication time possessed a positive impact whilst the S_mix_*sonication time possessed a negative impact on % transmittance. The ANOVA analysis and model summary statistics for the experimental results exhibited a greater R^2^ of 0.9949 for quadratic Equation (2).

Equation (3) and Figure 4C displayed the impact of the oil concentration, S_mix_ concentration, and sonication time on %CDR. The %CDR was enhanced with the increase in the concentration of S_mix_ and sonication time, whereas it was reduced with an increase in the concentration of the oil. The combination of the oil*S_mix_, oil*sonication time, and S_mix_*sonication time possessed a negative effect on %CDR (Figure 4(C1–C3)). These results depict that oil is the main influencing independent variable over S_mix_ and sonication time because a small increase in the percentage of oil causes more of an increase in the droplet size, leading to a hindrance in the drug release. The ANOVA analysis and model summary statistics for the experimental results showed a greater R^2^ of 0.9974 for quadratic Equation (3).

The main objective of the CCD-based optimization was to obtain the optimal formulation components to prepare the nanoemulsion. Hence, to obtain it, constraints were applied to the dependent as well as to the independent variables. In-range was selected as the constraint for all the independent variables, whereas the maximum was the constraint for the % transmittance and %CDR and the minimum for the droplet size. Then, the CCD provided the optimized recipe to formulate the formulation and predicted that the formulation must have the oil, S_mix_, and sonication time of 1.25%, 15.73%, and 90 s, respectively, to obtain the best results for the droplet size, %transmittance, and %CDR of 69.94 nm, 95.10%, and 92.00%, respectively. Then, the formulation suggested by the design was prepared, and it was found that the results of the dependent variables were very close to the results suggested by the design (Table 4).

### 2.5. Characterization of Optimized Formulation

#### 2.5.1. Droplet Size and PDI Determination

**Figure 5:**
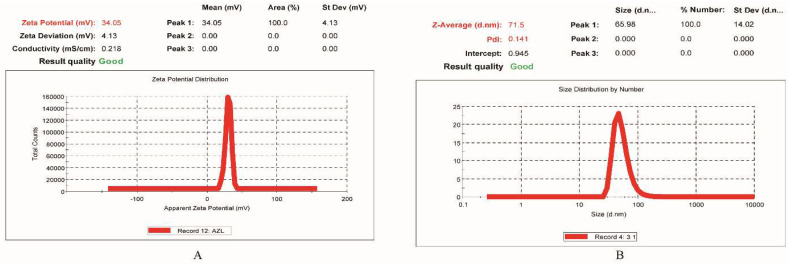
(**A**) depicts the droplet size as well as PDI and (**B**) depicts the zeta potential of the optimized AZL-NE formulation.

The optimized AZL-NE formulation exhibited an average droplet size of 71.5 nm (Figure 5A), which is less than 100 nm, showing that the optimized AZL-NE formulation was better in terms of droplet size and that the smaller droplet size of the nanoemulsion guarantees a better permeation of the drug, resulting in an enhanced surface area. The PDI of the optimized AZL-NE formulation was found to be 0.141 (Figure 5A), which showed the narrow size distribution of the droplets in the nanoemulsion formulation. The value of the PDI lies in the range from 0 (perfectly even sample) to 1 (high polydisperse sample), and a PDI value of less than 0.3 is acceptable in the case of nano-formulations [17].

#### 2.5.2. Surface Charge (Zeta Potential) Determination

The optimized AZL-NE formulation exhibited a zeta potential of 34.05 mV (Figure 5B). The higher value for the zeta potential provides superior repulsion between the droplets in the formulation, leading to a reduction in the chances of the aggregation of the droplets [18]. Moreover, the droplets in a formulation with a zeta potential of higher than +30 mV or −30 mV are considered stable, and hence, the optimized AZL-NE formulation can be considered a stable formulation [19].

#### 2.5.3. pH, Viscosity, and Refractive Index Determination

The optimized AZL-NE formulation exhibited a pH of 6.68 ± 0.22, which lies in the pH range for human mucosal tissues (5–6.5), supporting the non-irritant nature of the formulation. The isotropic nature of the formulation was validated by the refractive index, which was found to be 1.413 ± 0.03 for the optimized AZL-NE formulation. The viscosity of the optimized AZL-NE formulation was found to be 28.17 ± 0.52 cps, which is very low. Viscosity is a crucial parameter for deciding on the stability and efficacious drug release from the nanoemulsion [20].

#### 2.5.4. Percentage Transmittance Determination

%transmittance was investigated to assess the droplet size as well as the stability of the formulation because any change in %transmittance leads to a change in droplet size as well as the size distribution of the formulation [21]. The optimized AZL-NE formulation was found to be 93.46 ± 1.13%, which is close to 100, implying that the formulation was transparent, clear, and able to transmit the light.

#### 2.5.5. Drug Content Determination

The optimized AZL-NE formulation exhibited a drug content of 96.98 ± 0.94%, depicting a higher drug loading capability of the formulation, which is an essential characteristic for nanoemulsion.

#### 2.5.6. Surface Morphology Study Using Transmission Electron Microscopy (TEM)

**Figure 6:**
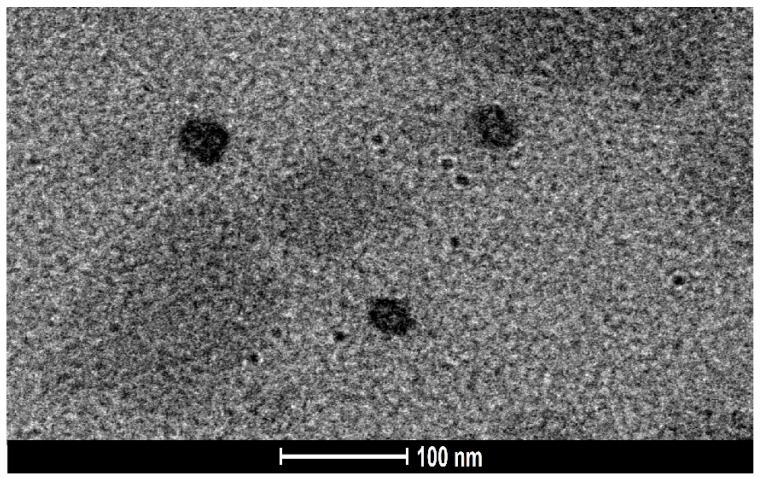
Surface morphology (TEM) image of optimized AZL-NE formulation.

The results of the surface morphology study using TEM are depicted in Figure 6, which reveals that the droplets of the optimized AZL-NE formulation possess a spherical shape, with a size of less than 100 nm. Moreover, no aggregation was present between the droplets of the formulation, which reflects the stability of the formulation. The results of the TEM analysis were in agreement with the droplet size of the formulation determined by the zeta sizer.

#### 2.5.7. Endorsement of Molecular Dispersion of Drug in Nanoemulsion Using Differential Scanning Calorimetry (DSC)

**Figure 7:**
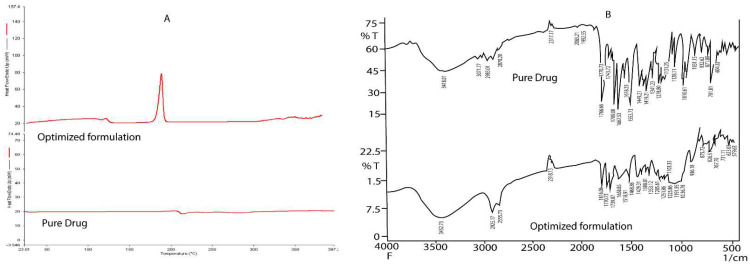
(**A**,**B**) depict DSC thermograms and FTIR spectrum for pure drug and optimized AZL-NE formulation, respectively.

Figure 7A depicts the DSC peaks for AZL and the optimized AZL-NE formulation. The DSC peak for AZL was obtained at 213.390 °C, which lies within the reference values of 212–214 °C, signifying its purity and crystalline nature. However, the peak of the optimized AZL-NE formulation was obtained at 170.914 °C, which is the peak of the mannitol used as a cryoprotective agent in the lyophilization process of the optimized AZL-NE formulation. The peak of AZL disappeared in the optimized AZL-NE formulation. This confirmed that AZL was completely solubilized in the nanoemulsion and that the crystallization of AZL did not occur during the preparation of the nanoemulsion, signifying the amorphous nature of AZL in the formulation. Apart from this, the DSC peaks demonstrated that AZL and the excipients in the optimized AZL-NE formulation did not have any chemical interaction.

#### 2.5.8. Drug–Excipient Interactions Assessment Employing Fourier Transform Infrared Spectroscopy (FTIR)

FTIR analysis was performed to analyze the physiochemical interactions between the different ingredients of the formulation and the drug. The results of the FTIR spectrum of the pure drug and optimized AZL-NE formulation are depicted in Figure 7B. The main peaks of the FTIR spectrum of the pure azilsartan medoxomil were at 3448.01 cm^−1^ (N-H stretching); 3071.77 cm^−1^ (C-H stretching); 1823.77 cm^−1^ (C=O stretching); 1709.00 cm^−1^ (C=O stretching); 1667.53 cm^−1^ (C=O stretching); 1553.73 cm^−1^ (C=C stretching); and 1251.86 cm^−1^ (C-O stretching). The FTIR spectrum of the optimized AZL-NE formulation showed peaks at 3452.73 cm^−1^ (N-H stretching); 2925.17 cm^−1^ (C-H stretching); 1816.06 cm^−1^ (C=O stretching); 1739.87 cm^−1^ (C=O stretching); 1658.85 cm^−1^ (C=O stretching); 1598.91 cm^−1^ (C=C stretching); and 1278.80 cm^−1^ (C-O stretching). There was no major deviation in peaks of the FTIR spectrum for the optimized AZL-NE formulation and pure azilsartan medoxomil drug, which confirmed the absence of molecular interaction between the drug and the formulation components. All the peaks in the FTIR spectrum of the pure drug and the optimized AZL-NE formulation were present as per the functional groups present in the structure of the drug.

#### 2.5.9. In Vitro Drug Release Studies Employing Dialysis Membrane

**Figure 8:**
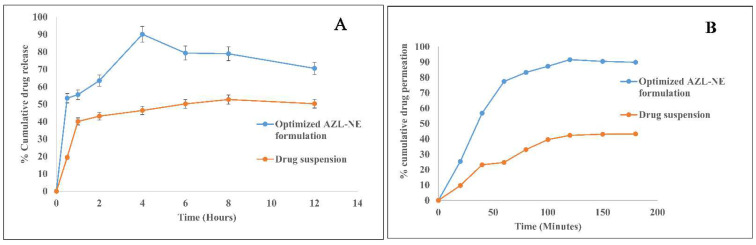
In vitro drug release comparison of optimized AZL-NE formulation with drug suspension (**A**) and ex vivo intestinal permeation comparison of optimized AZL-NE formulation with a drug suspension (**B**).

In vitro drug release studies were carried out to compare the release of AZL from the optimized AZL-NE formulation to the respective suspension (Figure 8A). The optimized AZL-NE formulation exhibited a drug release of 53.41 ± 1.26% in the first 30 m, whereas it was only 19.40 ± 0.64% for the drug suspension at the same time. The optimized AZL-NE formulation provided a maximum drug release of 90.14 ± 0.94% in 4 h, followed by deceleration [22], whilst the drug suspension provided a maximum drug release of 52.65 ± 0.35% in 8 h. This 1.71-fold increase in the release of AZL from the optimized AZL-NE formulation compared to its suspension could be attributed to the presence of the drug in solution form, owing to the reduced droplet size which will provide an increased surface area for drug dissolution, and an increase in the surface area leads to an increase in the dissolution rate, as suggested by the Noyes–Whitney equation [23]. The drug release of the drug suspension being poorer than the optimized nanoemulsion formulation is attributed to the low aqueous solubility of the drug, the coarse particle size, and the presence of the drug in crystalline form. As a result, the optimized AZL-NE formulation boosted the solubility of the drug as well as the dissolution rate due to the reduced droplet size of the nanoemulsion formulation.

#### 2.5.10. Ex Vivo Intestinal Permeation Studies

Intestinal permeation studies were evaluated to know the permeation and transit of AZL from the optimized AZL-NE formulation, which was contrasted with the AZL suspension (Figure 8B). It was found that the optimized AZL-NE formulation provided a burst of permeation of 77.47% in one hour, whereas the suspension provided only 24.76% at the same time. The optimized AZL-NE formulation exhibited a maximum %cumulative drug permeation of 91.65% in 2 h, whilst the drug suspension exhibited a maximum %cumulative drug permeation of 43.32% in 3 h. The apparent permeability (Papp) for the optimized AZL-NE formulation was 2.32 × 10^−4^ cm/s, which was significantly greater than the Papp of the drug suspension of 1.07 × 10^−4^ cm/s. A 2.16-fold increase in apparent permeability through the intestinal membrane could be attributed to an increase in the aqueous solubility of AZL, owing to the nano-size of the optimized AZL-NE formulation; an increase in the aqueous solubility ensures the availability of more drug in a solution form, which will increase the permeation of drug through the intestinal membrane due to its lipoidal nature. Apart from this, the presence of oil improves the permeation of the drug through the lipoidal membrane. The presence of ethyl oleate as a surfactant and Transcutol P is also responsible for the improvement in the permeation of the drugs through the intestinal membrane, owing to their penetration-enhancing properties. Moreover, ethyl oleate is responsible for the improvement in drug absorption due to the inhibition of the P-gp pump present in the enterocytes of the gastrointestinal tract by ethyl oleate [24].

#### 2.5.11. Estimation of the Depth of Permeation Using Confocal Laser Scanning Microscopy (CLSM)

**Figure 9:**
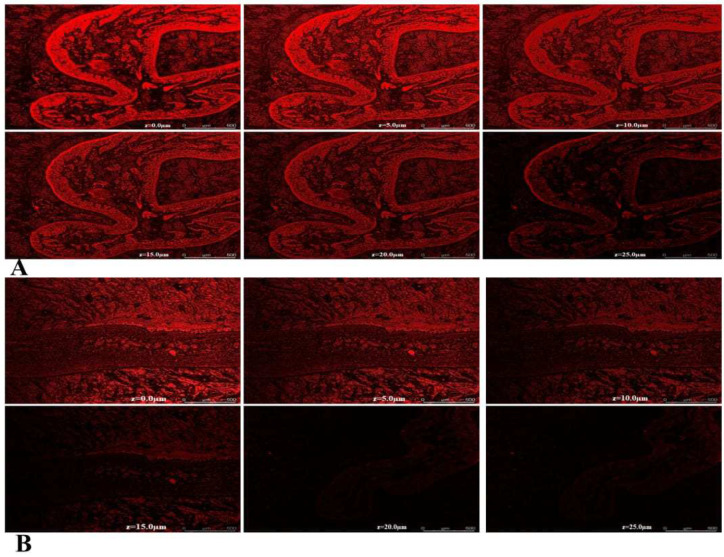
Image of CLSM for intestinal tissues treated with AZL-NE (**A**) and drug suspension (**B**).

CLSM studies were performed to assess the transport of the optimized AZL-NE formulation and the AZL suspension across the enterocytes using rhodamine-B dye. The intensity and depth of rhodamine-B across the enterocytes were determined by observing the intestinal tissues at the Z axis. The depth of the rhodamine-B fluorescence was observed up to 25 µm in the case of the optimized AZL-NE formulation (Figure 9A), whilst it was decreased to 10 µm in the case of the AZL suspension (Figure 9B), signifying a 2.5- fold enhancement in the permeation of AZL from the optimized AZL-NE formulation compared to its suspension. This enhancement in permeation is possible due to the nano-size of the droplets in the nanoemulsion formulation. Apart from this, previous studies have reported that surfactants with an HLB value of 10–17 improve the absorption and permeability of drugs by inhibiting the P-gp efflux pump [25]. Hence, in this research, the improved permeation of AZL from the optimized AZL-NE formulation might be due to the presence of tween 80 with an HLB value of 15. Moreover, the improved permeation could also be due to the presence of Transcutol P as a co-surfactant.

#### 2.5.12. Assessment of Stability using Thermodynamic Stability and Storage Stability

The major issue with nanoemulsion formulation is maintaining its stability throughout the shelf life. Hence, to verify it, stability testing was performed. The results of the thermodynamic stability studies demonstrated that there was no phase separation on the exposure of the optimized AZL-NE formulation to heating–cooling cycles, centrifugation tests, and freeze–thaw cycles. However, minor creaming in the optimized formulation was observed, which was redistributed after minor shaking of the formulation.

The results of the storage stability studies demonstrated that the optimized AZL-NE formulation has no sign of phase separation on visual inspection when stored in different storage conditions. The change in droplet size, % transmittance, and %CDR was minor for the optimized AZL-NE formulation when stored at 25 ± 1 °C and 4 ± 1 °C. The data generated after the storage stability studies for the optimized AZL-NE formulation are summarized in Table 5, depicting a better storage stability for the optimized AZL-NE formulation.

## 3. Materials and Methods

### 3.1. Materials

Azilsartan medoxomil was obtained from Alkem laboratories limited, Mumbai, India as a gift sample. Almond oil, olive oil, rose oil, semsam oil, cod liver oil, coconut oil, castor oil, Nigella sativa oil, corn oil, sunflower oil, tween 20, tween 60, tween 80 were purchased from SD Fine chemicals (Mumbai, India). Ethyl oleate, oleic acid, span 80, labrasol, Labrafil^®^ M 1944 CS, kolliphore HS, and Transcutol P were obtained as gift samples from Gattefosse, Mumbai, India. PEG 200, PEG 300, PEG 400, and propylene glycol were procured from Acros organic, Mumbai, India. Potassium dihydrogen phosphate and disodium hydrogen phosphate, rhodamine B, methanol, and mannitol were procured from Sigma-Aldrich (New Delhi, India). All the chemicals used for experimentation were of analytical grade.

### 3.2. Methods

#### 3.2.1. Selection of Formulation Components Using Solubility Determination

The solubility of azilsartan medoxomil was determined in various formulation components, namely oils, surfactants, and co-surfactants, to select the best materials required for the preparation of nanoemulsion. It was carried out in a variety of oils (almond oil, olive oil, rose oil, semsam oil, cod liver oil, coconut oil, corn oil, sunflower oil, ethyl oleate, castor oil, oleic acid, and Nigella sativa oil), surfactants (tween 20, tween 60, tween 80, span 80, labrasol, Labrafil^®^ M 1944 CS and kolliphore HS 15) and co-surfactants (PEG 200, PEG 300, PEG 400, Transcutol P, and propylene glycol) by dissolving the excess quantity of the drug in 2 mL of various oils, surfactants, and co-surfactants separately in 2.5 mL eppendrof tubes, followed by continuous vortexing for 48 h at room temperature to attain the equilibrium. Then, the samples were centrifuged at 3000 rpm for 15 min, and the supernatant was collected. The drug was extracted from the supernatant using methanol, and analysis was carried out by UV Spectrophotometer (UV 1700, Shimadzu, Japan) [26,27].

#### 3.2.2. Miscibility Determination of Oil, Surfactant, and Co-Surfactant

The miscibility of the oil phases was determined with surfactants and co-surfactants selected on the basis of the solubility profile by mixing at the ratio of 1:1, followed by vortexing for approximately 15 min. Then, the samples were kept without any disturbance for 24 h and evaluated visually for phase separation or any visible color changes. The mixtures that appeared clear were selected for further studies [28].

#### 3.2.3. Pseudo-Ternary Phase Diagram Studies

The main purpose of the pseudo-ternary phase diagram studies is to identify the optimized ratio of the selected surfactant and co-surfactant required for the preparation of nanoemulsion [29]. Different ratios (1:1, 1:2, 1:3, 1:4, 2:1, 3:1, and 4:1) of surfactant and co-surfactant (S_mix_) were mixed with selected oil in different ratios of 9:1, 2:8, 3:7, 4:6, 5:5, 6:4, 7:3, 8:2, and 9:1, followed by titration of each mixture of oil and Smix against the deionized water under continuous stirring. After each addition of the deionized water to the mixture, visible observation for clarity and turbidity was performed. Those systems that seemed transparent and easily flowable were marked on a pseudo-three-component phase diagram representing the aqueous phase, the second representing the oil, and the third representing the S_mix_ ratio, utilizing the CHEMEX School software ver 3.51 (Arne Standnes, USA) [30].

#### 3.2.4. Preparation of Drug-Loaded Nanoemulsion

AZL-loaded nanoemulsion was prepared using the ultrasonication method [13,31]. In this method, the fixed amount of the drug was filled into the glass vial with the required amount of the oil, followed by the dissolution of the drug in the oil along with the continuous addition of S_mix_. After that, the obtained mixture was micro-titrated with distilled water to provide an emulsion with coarse droplets. To convert the coarse emulsion into nanoemulsion, ultrasonication was employed for 30–90 s, using the ultrasonic processor of 30 kHz (Hielscher-Ultrasound Technology, Germany) [21]. Ultrasonication may cause cavitation, resulting in heat production; hence, the sonication was performed in seconds [32].

#### 3.2.5. Optimization of Formulation Components for Nanoemulsion

Central composite design (CCD) was employed to optimize the main components required for the preparation of the nanoemulsions, using Design Expert software, Stat-Ease, Minneapolis, MN, USA [13]. The optimization was carried out using oil (%), S_mix_ (%), and sonication time (S) as independent variables, depending upon their potential to influence the dependent variables, namely droplet size (Y1; nm), %transmittance (Y2; %), and %CDR (Y3; %). Amongst the independent variables, oil (%) and Smix (%) were formulation variables and sonication time (s) was the process variable, which varied by three concentrations (high, medium, and low), as suggested by pseudo-ternary phase diagram studies [33]. The design suggested 17 formulation runs amongst 3 central points, 8 factorial points, and 6 axial points. The independent variables and dependent variables along with their levels are summarized in Table 6.

#### 3.2.6. Characterization of Optimized Formulation

##### Droplet Size and Polydispersity Index (PDI) Determination

The droplet size of nanoemulsion is a crucial characteristic as smaller particles will produce more surface area, which will eventually improve the drug’s absorption. The droplet size and PDI of the optimized AZL-NE formulation were determined using the dynamic light scattering technique (Zetasizer 1000 HAS, Malvern Instruments, Malvern, UK). The procedure involved the dilution of the formulation ten times with double distilled water; it was placed in a quartz cuvette for the determination, at an angle of 90° and a temperature maintained at 25 °C [34].

##### Surface Charge (Zeta Potential) Determination

The surface charge or zeta potential of the optimized AZL-NE formulation was determined using the Zetasizer 1000 HAS, Malvern Instruments, UK. The procedure involved the dilution of the formulation ten times with double distilled water; it was placed in a quartz cuvette for the determination of the surface charge [34].

##### pH, Viscosity, and Refractive Index Determination

The pH of the formulation was determined at 25 ± 0.5 °C, using a previously calibrated digital pH meter (Mettler Toledo, Langacher, Switzerland). The viscosity was determined by a Brookfield viscometer (DV-III+ Rheometer, Brookfield, WI, USA), employing a cone and plate at a temperature of 25 °C and speed of 10 rpm [35]. The refractive index was determined using an Abbe refractometer(Nirmal International, India) at room temperature [36].

##### Percentage Transmittance Determination

Percentage transmittance indicates the clarity of the formulation. It was determined spectrophotometrically using a UV-VIS spectrophotometer (Shimadzu, Japan). In this, 1 mL of the formulation was diluted 100 times with distilled water, followed by analysis at the wavelength of 247 nm [37].

##### Drug Content Determination

The drug content of the optimized AZL-NE formulation was determined by dissolving 1 mL of formulation in a suitable quantity (10 mL) of methanol. This mixture was then shaken for 30 min at 50 rpm at 37 ± 0.5 °C in an incubator (LSI-2005 RL, Lab Tech Co., Seoul, Korea), followed by the collection of the supernatant liquid after 30 min; it was then analyzed using a UV spectrophotometer (Shimadzu, Japan) at 247 nm and methanol as a blank [38].

##### Surface Morphology Study Using Transmission Electron Microscopy (TEM)

The surface morphological characterization of the optimized AZL-NE formulation was performed using transmission electron microscopy (TEM) (Tecnai G20 HR-TEM. Thermo Scientific, Waltham, MA, USA). It was performed by placing one drop of formulation on wax paper and then transferring it to the copper grid (300 mesh), stained with 2% *w*/*v* phosphotungstic acid, followed by air drying. Then, the dry sample was displayed under the transmission electron microscope to evaluate the size and shape of the droplets of the formulation [18].

##### Endorsement of Molecular Dispersion of Drug in Nanoemulsion Using Differential Scanning Calorimetry (DSC)

The DSC thermograms of the pure drug and optimized formulation were obtained using DSC Perkin Elmer. To obtain the thermogram of the optimized formulation, the lyophilization of the formulation was performed using mannitol as a cryoprotective agent at a concentration of 5% due to the possession of higher solubility in water and also the conversion into the crystalline form on freeze-drying. To obtain the DSC thermograms, 2 mg of pure drug and lyophilized powder of formulation were sealed in an aluminum pan separately. Then, the DSC was performed over the temperature of 30 to 300 °C, along with a heating rate of 10 °C/min in an air environment. The flow of nitrogen gas was maintained at 60 mL/min [21].

##### Drug–Excipient Interactions Assessment Employing Fourier Transform Infrared Spectroscopy (FTIR)

The FTIR analysis of the pure drug and the optimized formulation was carried out employing the potassium bromide pellet technique. In this technique, a homogenous mixture was made by adding samples of pure drug and the lyophilized form of the optimized formulation separately with potassium bromide at a ratio of 1:10, followed by the grinding of the mixture. Then, a small amount of powdered mixture was compressed to a thin, semitransparent film (pellets) by the application of pressure. The pellet’s IR spectrum was assessed between 500 and 4000 cm^−1^ using the FTIR spectrophotometer [21].

##### In Vitro Drug Release Studies Employing Dialysis Membrane

Drug release studies were implemented to compare the drug release of the optimized AZL-NE formulation with a drug suspension. In this, one milliliter of optimized AZL-NE formulation and drug suspension was filled into the separate dialysis bag, which was then dipped into the beaker with 80 mL of simulated intestinal fluid of pH 6.8 [18]. The beaker was kept in a magnetic stirrer, which was operated at 150 rpm and 37 ± 0.5 °C. At scheduled time intervals (30, 60, 120, 240, 360, 480, and 720 min), an aliquot of 5 mL was collected from the beaker, followed by the addition of fresh dissolution medium to the beaker to maintain the sink conditions. The collected samples were carried out for analysis using a validated UV-spectrophotometer (UV 1700, Shimadzu, Japan) at a wavelength of 247 nm [39]. The cumulative drug release from the optimized AZL-NE formulation and drug suspension was determined using the formula:

(4) $$\%CDR=\frac{DR}{IDC}\times 100$$

The obtained values were plotted as %CDR versus time.

##### Ex Vivo Intestinal Permeation Studies

Ex vivo intestinal permeation studies of the optimized AZL-NE formulation and drug suspension were performed using the non-inverted rat sac model [40]. In this study, a female Wistar rat weighing between 100 and 150 g was kept in standard conditions with free access to clean drinking water. The ileum portion of the small intestine was excised and cleaned of facial debris using Tyrode’s solution. The cleaned section of the small intestine was converted into the sac, tying one end firmly using thread, followed by insertion of the optimized AZL-NE formulation and drug suspension separately at a defined concentration in the sac and then also tying another end firmly using thread. The sac was then placed in a jacketed glass with 40 mL of Tyrode’s buffer (dissolution medium, pH 6.8) which had been previously warmed to 37 ± 0.5 °C and oxygenated for 120 min with 95% oxygen through an aerator. At specified time intervals (20, 40, 60, 80, 100, 120, 150, and 180 min), a sample of 4 mL was collected, followed by the addition of a fresh dissolution medium to maintain the sink conditions. All the collected samples were filtered through the membrane filter with a pore size of 0.45 µm and then analyzed using the UV spectrophotometer. The amount of the drug that escaped within 180 min from the optimized AZL-NE formulation was compared to the drug suspension and plotted as % cumulative drug permeation versus time [41]. The apparent permeability (*P_app_*) was determined using the formula:

(5) $$P_{app}=\left(\frac{dQ}{dt}\right)/\;\left(A\times Ci\right)$$

where (*dQ*/*dt*), *A*, and *Ci* denote the rate of drug permeation outside the sac, the surface area of the sac, and the initial concentration into the sac, respectively.

##### Estimation of the Depth of Permeation Using Confocal Laser Scanning Microscopy (CLSM)

CLSM is a useful method for increasing the visibility, penetration, and depth of nano-formulation in various tissues. Using optical laser scans produces deeper-selective, higher-resolution images of the tissue or the gut that are superior to fluorescence or optical microscopy. In this study, a female Wistar rat was used and sacrificed using cervical dislocation. Then, 4 cm-long ileum portions were excised from the small intestine followed by cleaning using a normal saline solution to remove all facial debris. The intestinal sacs were filled with a sample of rhodamine B-labeled optimized AZL-NE formulation and rhodamine B-labeled drug suspension using the syringe, followed by the tying of both ends of the intestinal sac firmly using thread. These intestinal sacs were kept in Tyrode’s buffer at the temperature, speed, and oxygen supply of 37 ± 0.5 °C, 45 rpm, and 95%, respectively, for 3 h [42]. The intestinal sacs were then cut lengthwise into min species and inserted into slits after being washed with saline solution to remove any remaining free rhodamine dye. The depth of permeation of the rhodamine B across the z-axis of the intestinal wall was estimated using confocal laser scanning microscopy (Leica Microsystems SP8, Mumbai, India) [18].

##### Assessment of Stability Using Thermodynamic Stability and Storage Stability Studies

The stability and phase reliability of the optimized AZL-NE formulation was evaluated using thermodynamic stability studies under varying conditions of temperature and centrifugal force. Under the thermodynamic stability studies, three distinctive tests were performed, namely heating–cooling cycles, centrifugation tests, and freeze–thaw cycles. In the heating and cooling cycles, the formulation was conducted in six cycles at 4 °C and 45 °C for 48 h each, followed by observation for phase separation. After this, a centrifugation test was performed in which the formulation was subjected to centrifugation at 3500 rpm for 30 min, followed by observation for phase separation. Then, freeze–thaw cycles were carried out, in which the formulation was subjected to six cycles of −21 °C and 25 °C for 48 h each and observed for phase separation [43].

The storage stability of the optimized AZL-NE formulation was assessed for one month, during which the formulation was packed in glass vials (amber colored) and stored at different conditions of temperature (25 ± 1 °C, room temperature, and 4 ± 1 °C, refrigeration), followed by an examination of the formulation at a specified time interval (10, 20, and 30 days) for phase separation, creaming, droplet size, % transmittance, and %CDR [39].

## 4. Conclusions

AZL-loaded nanoemulsion was prepared and optimized successfully using a central composite design to improve the aqueous solubility and intestinal permeation of the drug. The optimized AZL-NE formulation exhibited the droplet size, % transmittance, and %CDR of 71.5 nm, 93.46 ± 1.13%, and 90.14 ± 0.94%, respectively. The optimized AZL-NE formulation provided a drug release that was 1.71 times higher than the drug suspension. The drug permeation from the optimized AZL-NE formulation was 2.1-fold greater than the drug suspension through the intestinal segment of a rat. The results of CLSM also showed a deeper penetration of the optimized AZL-NE formulation than the drug suspension. All these results showed the superiority of nanoemulsion in the improvement of the aqueous solubility and intestinal permeability of hydrophobic drugs. The observed results opened the door for additional research, in which pharmacokinetics and pharmacodynamics studies will be conducted to approve the bioavailability and in vivo potential of the drug. In conclusion, this study effectively optimized AZL-NE via a central composite design, which demonstrates the prospective potential for improving intestinal permeability and may help to improve the oral bioavailability of azilsartan medoxomil.

## Figures and Tables

**Table 1:** Results of miscibility determination of selected oils with selected surfactants and co-surfactants.

Oils	Codes	Surfactant	Co-Surfactant	Miscibility Outcomes
Nigella sativa oil	1	**Tween 80**	**Transcutol P**	**Clear**
	2	Tween 80	PEG 300	Phase Separation
	3	Tween 80	PEG 400	Phase Separation
Cod liver oil	4	Tween 80	Transcutol P	Phase Separation
	5	Tween 80	PEG 300	Phase Separation
	6	Tween 80	PEG 400	Phase Separation
Ethyl oleate	7	**Tween 80**	**Transcutol P**	**Clear**
	8	Tween 80	PEG 300	Phase Separation
	9	Tween 80	PEG 400	Phase Separation
	10	**Labrasol**	**Transcutol P**	**Clear**
	11	**Labrasol**	**PEG 300**	**Clear**
	12	Labrasol	PEG 400	Phase Separation
Nigella sativa oil	13	Labrasol	Transcutol P	Phase Separation
	14	Labrasol	PEG 300	Phase Separation
	15	**Labrasol**	**PEG 400**	**Clear**
Cod liver oil	16	Labrasol	Transcutol P	Phase Separation
	17	Labrasol	PEG 300	Phase Separation

**Table 2:** CCD-observed AZL-NE formulation experimental runs along with experimental values and predicted values for dependent variables.

Formulations	Type of Point	Independent Variables			Dependent Variables					
					Experimental Values			Predicted Values		
Run	Point Type	A: Oil (%)	B: S_mix_ (%)	C: Sonication Time (S)	Droplet Size (nm) Y1	%Transmittance (%) Y2	%Drug Release (%) Y3	Droplet Size (nm) Y1	%Transmittance (%) Y2	%Drug Release (%) Y3
1	Factorial	2.5	8.75	30	311.9	74.67	23.03	303.96	74.55	22.87
2	Axial	2.92612	13.125	60	221.92	83.46	23.08	227.27	83.31	22.64
3	Factorial	2.5	17.5	90	144.21	90.43	33.31	134.24	91.15	33.29
4	Central	1.875	13.125	60	94.83	91.65	57.23	96.57	91.81	58.48
5	Factorial	1.25	8.75	90	107.04	88.56	68.87	109.00	88.41	68.28
6	Axial	1.875	13.125	110.454	85.21	91.76	62.87	78.79	91.65	62.94
7	Factorial	1.25	17.5	90	80.69	93.89	91.08	84.91	94.08	92.33
8	Axial	0.823879	13.125	60	85.04	95.3	94.92	84.95	95.35	93.82
9	Factorial	1.25	8.75	30	177.78	86.57	47.38	184.03	85.92	48.49
10	Axial	1.875	5.76716	60	266.74	72.88	22.06	257.91	73.77	22.30
11	Axial	1.875	13.125	9.54622	144.38	87.91	51.52	156.06	87.92	49.90
12	Factorial	1.25	17.5	30	91.02	92.76	77.12	75.73	93.34	78.09
13	Axial	1.875	20.4828	60	95.01	91.85	56.67	109.10	90.86	54.89
14	Factorial	2.5	17.5	30	191.32	78.74	24.02	202.89	78.23	24.14
15	Central	1.875	13.125	60	100.95	92.12	60.73	96.57	91.81	58.48
16	Factorial	2.5	17.5	30	156.76	88.98	35.89	151.08	89.20	37.57
17	Central	1.875	13.125	60	94.83	91.65	57.23	96.57	91.81	58.48

**Table 3:** Summarized regression analysis data for selected dependent variables.

Quadratic Model	R^2^ Value (Coefficient of Correlation)	Adjusted R^2^	Predicted R^2^	Adeq. Precision	Standard Deviation	Mean	Coefficient of Variation % (C.V.%)
Y1	0.9853	0.9664	0.8844	23.4079	12.71	144.10	8.82
Y2	0.9949	0.9883	0.9615	39.2628	0.7167	87.83	0.8167
Y3	0.9974	0.9940	0.9849	52.1242	1.79	52.18	3.43

**Table 4:** Predicted optimized formulation by design along with experimental results.

Batch	Independent Variables			Dependent Variables		
	A (%)	B (%)	C (Sec)	YI (nm)	Y2 (%)	Y3 (%)
Predicted	1.25	15.73	90	69.98	95.10	92.00
Observed	1.25	15.73	90	71.5	93.46 ± 1.13%	90.14 ± 0.94%

**Table 5:** Data of stability studies for optimized AZL-NE formulation.

Time (Days)	25 ± 1 °C (Room Conditions)				4 ± 1 °C (Refrigeration Conditions)			
	Phase Separation	Droplet Size (nm)	%Transmittance (%)	%CDR (%)	Phase Separation	Droplet Size (nm)	% Transmittance (%)	%CDR (%)
0	No	71.5	93.46 ± 1.13	90.14 ± 0.94	No	71.5	93.46 ± 1.13	90.14 ± 0.94
10	No	72.68	92.87 ± 0.29	89.48 ± 0.39	No	72.43	92.26 ± 0.65	89.53 ± 1.39
20	No	72.82	92.23 ± 0.42	89.41 ± 0.43	No	73.87	91.43 ± 0.87	88.98 ± 1.08
30	No	72.91	91.62 ± 0.34	88.74 ± 0.77	No	73.94	91.00 ± 0.71	88.78 ± 1.01

**Table 6:** Selected independent variables along with their levels and dependent variables.

Independent Variables	Levels				
	Low (−1)	Medium (0)	High (+1)	Axial (−1.68)	Axial (+1.68)
A = Oil (%)	0.62	1.285	1.67	0.193879	2.29612
B = S_mix_ (%)	4.36	8.74	13.12	1.37375	16.1063
C = Sonication time (s)	10	20	30	3.18207	36.8179
**Independent variables**	**Constraints**		**Importance**		
A = Oil (%)	In range		---------------		
B = S_mix_ (%)	In range		----------------		
C = Sonication time (Sec)	In range		-------------		
**Dependent variables**	**Constraints**		**Importance**		
Y1 = droplet size	Minimize		+++++		
Y2 = % transmittance	Maximize		+++		
Y3 = % cumulative drug release	Maximize		+++		

## Data Availability

Not applicable.
